# The relative abundance of wheat Rubisco activase isoforms is post-transcriptionally regulated

**DOI:** 10.1007/s11120-021-00830-6

**Published:** 2021-04-01

**Authors:** Juan Alejandro Perdomo, Peter Buchner, Elizabete Carmo-Silva

**Affiliations:** 1 Lancaster Environment Centre, Lancaster University, Lancaster, LA1 4YQ UK; 2 Plant Biology and Crop Science Department, Rothamsted Research, Harpenden, AL5 2JQ UK

**Keywords:** Rubisco, Rubisco activase, Gene expression, Protein abundance, Diel cycle

## Abstract

Diurnal rhythms and light availability affect transcription–translation feedback loops that regulate the synthesis of photosynthetic proteins. The CO_2_-fixing enzyme Rubisco is the most abundant protein in the leaves of major crop species and its activity depends on interaction with the molecular chaperone Rubisco activase (Rca). In *Triticum aestivum* L. (wheat), three Rca isoforms are present that differ in their regulatory properties. Here, we tested the hypothesis that the relative abundance of the redox-sensitive and redox-insensitive Rca isoforms could be differentially regulated throughout light–dark diel cycle in wheat. While *TaRca1-β* expression was consistently negligible throughout the day, transcript levels of both *TaRca2-β* and *TaRca2-α* were higher and increased at the start of the day, with peak levels occurring at the middle of the photoperiod. Abundance of TaRca-β protein was maximal 1.5 h after the peak in *TaRca2-β* expression, but the abundance of TaRca-α remained constant during the entire photoperiod. The redox-sensitive TaRca-α isoform was less abundant, representing 85% of the redox-insensitive TaRca-β at the transcript level and 12.5% at the protein level. Expression of Rubisco large and small subunit genes did not show a consistent pattern throughout the diel cycle, but the abundance of Rubisco decreased by up to 20% during the dark period in fully expanded wheat leaves. These results, combined with a lack of correlation between transcript and protein abundance for both Rca isoforms and Rubisco throughout the entire diel cycle, suggest that the abundance of these photosynthetic enzymes is post-transcriptionally regulated.

**Supplementary Information:**

The online version contains supplementary material available at 10.1007/s11120-021-00830-6.

## Introduction

Photosynthesis is one of the most important physiological processes in plants that begins with the absorption of light energy and leads to fixation of CO_2_ (Berry and Downton 1982). Photosynthesis is regulated by an internal timekeeping system, the circadian clock, which runs in a period of 24 h and regulates several molecular and physiological processes such as growth, enzyme activity and control of stomatal aperture (Harmer 2009). In this study, we set out to investigate whether the abundance of ribulose-1,5-bisphosphate carboxylase/oxygenase (Rubisco) and its molecular chaperone, Rubisco activase (Rca), change in concert with light availability throughout the diel cycle. Wheat (*Triticum aestivum* L.) is one of the most important crops worldwide, supplying more than 20% of the calories consumed by humanity (Ray et al. 2013). We have previously shown that wheat contains three Rca isoforms (Carmo-Silva et al. 2015) that differ in their regulatory properties (Perdomo et al. 2019). Here, we tested the hypothesis that the relative abundance of the three Rca isoforms in wheat leaves would change throughout the day, with a potential impact on Rubisco activity.

Rubisco consists of eight large subunits (LSU), encoded by a single gene (*rbcL*) in the chloroplast, and eight small subunits (SSU), encoded by a multigene family (*RbcS*), in the nuclear genome (Schmidt and Mishkind 1986; Roy 1989). Rubisco is the most abundant protein in plants and is responsible for net CO_2_ assimilation. However, Rubisco has been described as one of the most inefficient enzymes due to its very low catalytic turnover rate and the susceptibility to inhibition by unproductive binding of sugar-phosphate derivatives that lock the active sites in a closed conformation (Brooks and Portis 1988; Jordan and Chollet 1983; Portis 1995). Rubisco activase (Rca) is a catalytic chaperone of Rubisco belonging to the AAA+ protein family (Neuwald et al. 1999). Rca uses the energy from ATP hydrolysis to restore the catalytic competence of Rubisco by promoting the release of the inhibitory sugar-phosphates from Rubisco active sites (Portis 1995).

In many flowering plant species, Rca exists as two isoforms that are almost identical except for a 30–39 amino acid extension of the C terminus that is present only in the longer Rca-α isoform and differentiates it from the shorter Rca-β isoform (Salvucci et al. 1987; Werneke et al. 1989). Two cysteine residues at the C-terminal extension confer redox-sensitivity to Rca-α (Zhang and Portis 1999; Zhang et al. 2001). The Rca-α and Rca-β isoforms are the products of either alternative splicing or separate genes depending on the species. Three Rca isoforms, two β and one α, are encoded by two genes in wheat (Carmo-Silva et al. 2015). Expression of the *TaRca1* gene produces a Rca1-β isoform, whereas alternative splicing of the *TaRca2* gene produces either a Rca2-β or a Rca2-α isoform. The gene expression and abundance of Rca isoforms varies considerably among species, with Rca-α sometimes present in similar amount, but generally much less abundant than Rca-β (Salvucci et al. 1987, 2003; Yin et al. 2010).

*Rca* gene expression in higher plants is almost entirely restricted to green tissues, and is developmentally regulated by leaf age as well as regulated by light (Orozco and Ogren 1993; Watillon et al. 1993; Liu et al. 1996). The expression of *Rca* of numerous species including *Arabidopsis*, tomato, apple and rice is regulated by the circadian clock (Pilgrim and McClung 1993; Martino-Catt and Ort 1992; Watillon et al. 1993; To et al. 1999). In *Arabidopsis* and tomato, these circadian patterns are evident in the rate of net protein synthesis alongside with the accumulation of mRNA levels (Martino-Catt and Ort 1992; Pilgrim and McClung 1993). However, a physiological role for a circadian rhythm in Rca transcript accumulation is unclear and the level of Rca protein in mature tobacco leaves does not exhibit a similar oscillation (Klein and Salvucci 1995). It has been proposed that a physiological role in photosynthetic performance may exist in early stages of leaf development as Rca levels are low and *Rca* transcript abundance enhanced by circadian rhythm could impact protein abundance (Martino-Catt and Ort 1992; Pilgrim and McClung 1993).

Rubisco *rbcL* and *RbcS* mRNA amounts fluctuate during the diurnal cycle with different patterns in different species; however, only the *RbcS* subunit follows a circadian rhythm (Pilgrim and McClung 1993; Recuenco-Muñoz et al. 2015). In rice, *rbcL* and *RbcS* amounts fluctuated during diurnal cycle with peak abundance levels during the light phase of the photoperiod (Wang and Wang 2011). In *Arabidopsis* plants grown in a light/dark photoperiod, *RbcS* mRNA exhibits a diurnal pattern of expression, with peak abundance occurring soon after beginning of the light and minimum levels at the end of the light period (Pilgrim and McClung 1993). In contrast, the amount of *rbcL* and *RbcS* transcripts in *Chlamydomonas reinhardtii* were highest in the dark (Recuenco-Muñoz et al. 2015). Other studies with *C. reinhardtii* indicate that *rbcL* mRNA levels are not directly correlated to the amount of functional Rubisco protein (Winder et al. 1992; Cohen et al. 2006). This lack of correlation between gene expression and protein abundance suggests that the latter is post-transcriptionally regulated, and that transcript levels are insufficient to predict functional protein levels (Vogel and Marcotte 2012; Liu et al. 2016).

Despite the characterisation of Rca isoforms in wheat (Carmo-Silva et al. 2015; Perdomo et al. 2019) and gene expression studies under abiotic stresses such as drought and temperature (Zhao et al. 2017; Scafaro et al. 2019a; Degen et al. 2021) little is known about diurnal regulation of their abundance. The three wheat Rca activate Rubisco at different rates and differ in their sensitivity to light stimuli, changes in the ADP/ATP ratio (Perdomo et al. 2019). These findings suggest that changes in the relative abundance of each isoform could affect the rate of CO_2_ fixation by Rubisco in wheat leaves. The aim of this study was to characterise the expression levels of Rubisco and the three Rca isoforms present in wheat during the 24 h diel cycle and investigate whether the diurnal fluctuations in gene expression are translated into protein abundance. Likewise, we wanted to identify the time period when the protein amounts are highest and constant during the day.

## Materials and methods

### Plant material

Plants of *T. aestivum* L. cultivar Cadenza were grown from seed in 1.5 L pots (11 × 11 cm) containing Rothamsted description mix compost with 75% medium grade (L&P) peat, 12% screened sterilised loam, 3% medium grade vermiculite, 10% grit (5 mm screened, lime free). After 2 weeks, plants were thinned down to have 3 plants per pot, with a total of 90 plants (30 pots). Plants were grown in controlled environment cabinets (Fitotron Weiss Gallenkamp, UK) with an area of 1.68 m^2^ and growing height of 1.4 m. Plants were grown under a photoperiod of 16 h light with a PPFD of 500 μmol m^−2^ s^−1^; lights were on at 00:00 and off at 16:00 each day (Fig. S1). Air temperature was 20/18 °C day/night and the relative humidity was maintained at 60%. Plants were watered daily. Plants were grown in two controlled environment cabinets to have sufficient replication, 45 plants (15 pots) were placed in each cabinet and the plants in the two cabinets were planted and harvested in two consecutive days.

The experimental design was a split-plot in two blocks (i.e. two cabinets). Each sample was taken from a separate plant, and three samples were taken at each time point from each cabinet, resulting in six individual samples (i.e. six biological replicates) per time of the day, three from each of the two cabinets. The samples were taken throughout the diel cycle at 20:00, 22:00, 23:00, 24:00, 01:00, 02:00, 04:00, 06:00, 08:00, 10:00, 12:00, 14:00, 16:00, 17:00 and 18:00 h, with good representation of the period in darkness and in the light (Fig. S1).

The youngest fully expanded leaf with a visible collar, from the main tiller, was harvested 38 days after planting (growth stage Zadoks 2.5–3.0; Zadoks et al. 1974). The youngest fully expanded leaf was generally the fourth leaf, but in some more developed plants it corresponded to the fifth leaf. A section of 8 cm in the middle of the leaf, avoiding the leaf base and tip, was divided into four 2-cm-long segments and two of each of these used for each of two sub-samples: one for quantifying Rca and Rubisco protein abundance and another for gene expression analysis by qRT-PCR.

### Gene expression and quantification

Gene expression of Rca (*TaRca1-β*, *TaRca2-β*, *TaRca2-α*)*,* and Rubisco large and small subunits (*TarbcL* and *TaRbcS*), were determined by Real-Time Quantitative Reverse Transcription PCR (RT-qPCR). The extraction of total RNA from the wheat ~ 4 cm^2^ leaf samples was completed using a modified hot phenol method (Shinmachi et al. 2010; Verwoerd et al. 1989). The total RNA concentration and quality was determined by measuring the absorbance at 230, 260 and 280 nm with a Nanodrop spectrometer (Thermo Fisher Scientific, Inc., UK) and running on a 1% (w/v) agarose gel. A sub-sample of 1 μg of total RNA was used for cDNA synthesis, using Superscript III as per the manufacturer’s instructions (Life Technologies Ltd., UK).

For qRT-PCR a 1:10 dilution of cDNA was used with SYBRGreen (Platinum® SYBR® Green qPCR SuperMix-UDG w/ROX, Life Technologies, UK), in 25 μL reactions, as per the manufacturer’s instructions. Primer pairs specific to *TaRca1-β*, *TaRca2-β*, *TaRca2-α*, *TarbcL* and *TaRbcS* (primers were specific for *TaRbcS* located on chromosome 5A, 5B and 5D, group S3 as described by Degen et al. 2021), were used for qRT-PCR, alongside primers for two reference genes (Table S1). Primers were designed to bind to all three wheat sub-genomes, except for *rbcL*, which is encoded in the chloroplast genome. The qRT-PCR conditions were: 50 °C for 2 min, 95 °C for 10 min, followed by 40 cycles of 95 °C for 15 s and 60 °C for 1 min. Melt curves were also completed: 95 °C for 15 s, 60 °C for 1 min and 95 °C for 15 s (7500 Real-Time PCR machine, Applied Biosystems, Life Technologies, UK).

The mean primer efficiency was estimated using the linear phase of all individual reaction amplification curves (Ramakers et al. 2003) and calculated using the LinRegPCR package (Tuomi et al. 2010). The actin and succinate dehydrogenase genes were used as reference genes for the normalised relative quantification of expression. The normalised relative quantity (NRQ) of expression was calculated in relation to the cycle threshold (CT) values and the primer efficiency (*E*) of the target gene (*X*) and the normalising reference gene (*N*), based on Rieu and Powers (2009): NRQ = (*EX*)^−CT, *X*^/(*EN*)^−CT, *N*^.

### Rubisco and Rubisco activase protein quantification

To determine the amounts of Rubisco and Rca protein in the wheat ~ 4 cm^2^ leaf samples, extracts were prepared as described by Perdomo et al. (2018). Total soluble protein (TSP) concentration in the crude extracts was determined according to the Bradford (1976) method using bovine serum albumin as standard. Based on the TSP concentration all samples were diluted to 0.6 µg µL^−1^ and 3 µg of TSP was used per sample for Rubisco and Rca quantification. Proteins were separated by sodium dodecyl sulphate polyacrylamide gel electrophoresis (SDS-PAGE) on 12% gels hand casted and either visualised by staining with Coomassie Blue for Rubisco, or subject to immunoblotting for Rca. For the latter, proteins were transferred from the gel to a nitrocellulose membrane (iBlot, Thermofisher, UK), probed with a primary antibody anti-Rca produced in rabbit against cotton (Salvucci 2008) and a fluorescent secondary antibody for visualisation of Rca using an Odyssey Fc imaging (LI-COR, Lincoln, USA).

For Rca quantification, purified wheat Rca was used to load in each gel a series of four standards with increasing quantities, 0.01, 0.05, 0.1 and 0.15 µg, to prepare a calibration curve (Fig. S2). For Rubisco quantification, purified wheat Rubisco was used to load in each gel a series of four standards with increasing quantities, 0.1, 0.5, 1.0 and 1.5 µg, to prepare a calibration curve (Fig. S2).

### Data analysis and modelling

Data were analysed using R 3.6.2 (R Core Team 2020) and RSTUDIO 1.2.5033 (RStudio Team 2020), and graphs were prepared using the GGPLOT 2 package (Wickham 2017). Linear Mixed Effects Regression (LMER) was used to assess the significance of differences in the gene expression and protein abundance of Rca and Rubisco between the different sampling times. Mean values and standard error of the mean (SEM) are shown in figures. To estimate maximum gene expression and protein amounts for Rca and Rubisco, and the corresponding times at which the maximum values were reached, second- to fourth-order polynomials and generalised additive models (GAM) were fitted to the experimental data using the gam function from the MGCV 1.8-24 package in R (Wood 2017). The model that best-fit the experimental data was selected based on the Akaike information criterion (Akaike 1974) using the AIC function (Table S2). The ‘predict’ function was used to estimate the maximum gene expression and protein amount values and the corresponding times for each Rca isoform and Rubisco subunits. The standard error calculated for each model was used to predict 95% confidence intervals for each fit (dashed lines in the graphs).

## Results

### Gene expression of *TaRca2* and *TaRbcS* peaked at 8–10 h into the photoperiod

**Fig. 1:**
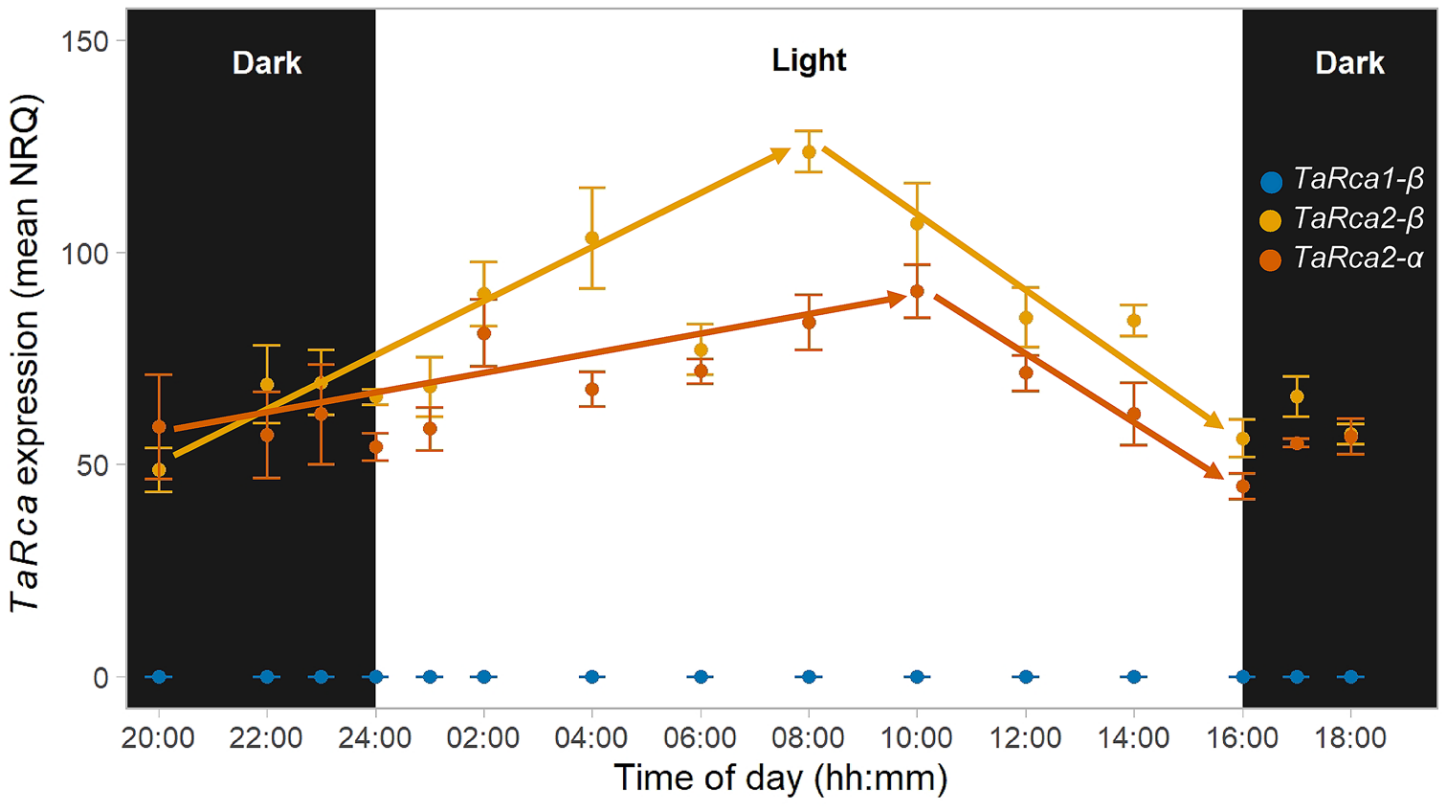
*TaRca1-β*, *TaRca2-β* and *TaRca2-α* expression throughout the diel cycle in young fully expanded leaves of wheat. Samples were taken from individual plants 38 days after planting (vegetative stage) at the indicated times during the night (“Dark”, black background) and day (“Light”, white background). Gene expression was estimated as normalised relative quantification (NRQ) using actin and succinate dehydrogenase as reference genes. Values are means ± SEM (*n* = 4–6 biological replicates). Arrows mean significant, positive and negative, correlations between gene expression and time of the day for *TaRca2β* and *TaRca2α* (Pearson correlation analysis, *P* < 0.01)

The expression of *TaRca1* and *TaRca2* encoding the three Rca isoforms present in wheat (*TaRca1-β*, *TaRca2-β* and *TaRca2-α*), as well as the Rubisco large (*rbcL*) and small (*RbcS*) subunits, were determined throughout the diel cycle using quantitative real-time PCR analysis. *TaRca2-β* and *TaRca2-α* transcripts were much more abundant than *TaRca1-β*, which was detectable in very small amounts. The expression of *TaRca2-β* was highest at the middle of the light phase, 8 h into the photoperiod, while *TaRca2-α* had the highest expression levels occurring 10 h into the photoperiod. The expression of both *TaRca2-β* and *TaRca2-α* decreased significantly in the second half of the light phase, reaching a minimum at the end of the light phase and remaining low and constant during the dark phase (Fig. 1)*.* The ratio between the *TaRca2-α* and *TaRca2-β* relative gene expression remained mostly constant throughout the diel cycle, with the expression of *TaRca2-α* corresponding to approximately 85 ± 3% relative to the *TaRca2-β* isoform (Fig. S3).

The relative expression of the *TaRca1* gene was extremely low, with Normalised Relative Quantity values (NRQ) from 0.001 to 0.008, which are much lower than its homologues encoded by the *TaRca2* gene. No significant differences in *TaRca1-β* expression were detected between time points throughout the diel cycle.

**Fig. 2:**
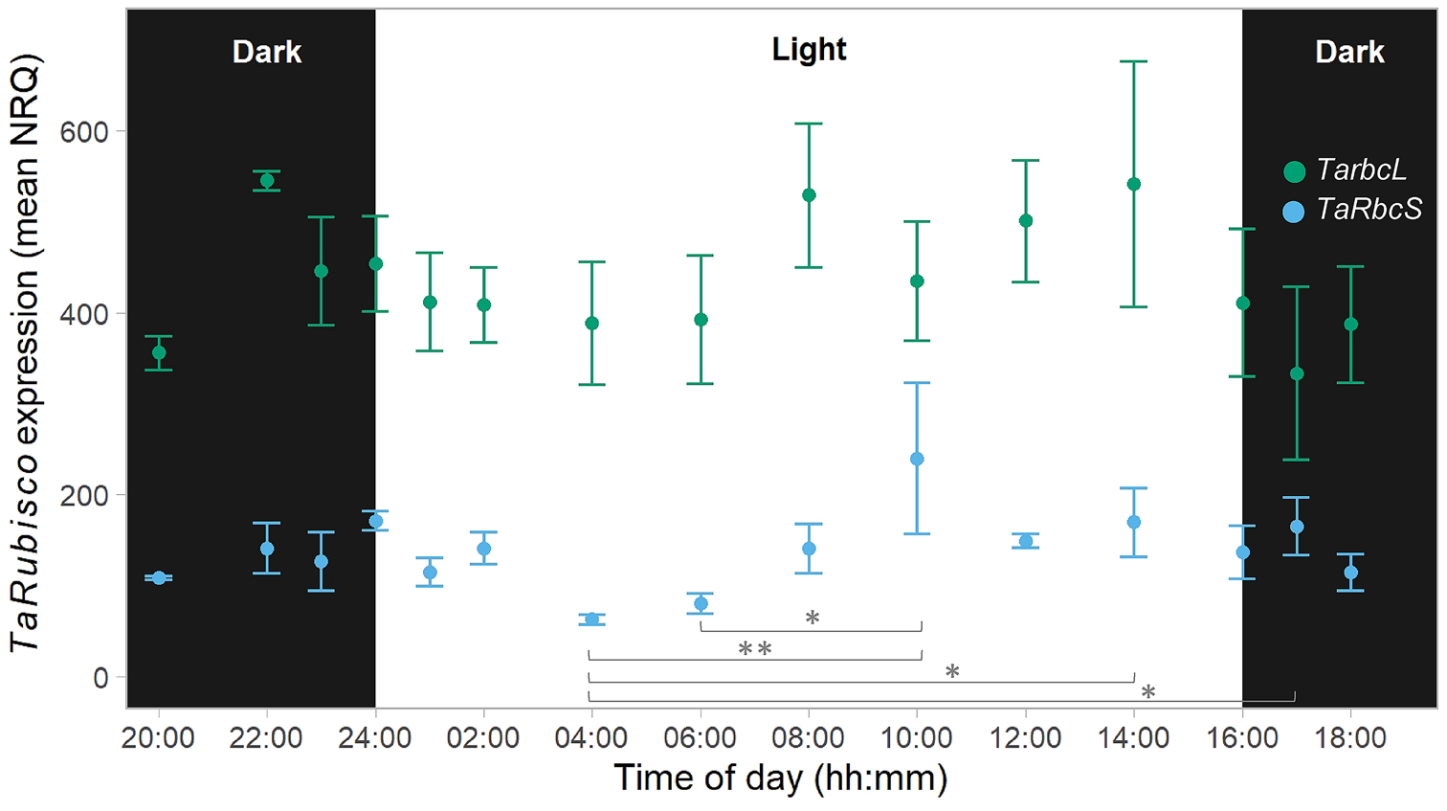
*TarbcL* and *TaRbcS* expression throughout the diel cycle in young fully expanded leaves of wheat. Samples were taken from individual plants 38 days after planting (vegetative stage) at the indicated times during the night (“Dark”, black background) and day (“Light”, white background). Gene expression was estimated as normalised relative quantification (NRQ) using actin and succinate dehydrogenase as reference genes. *TaRbcS* expression used primers specific to the genes in chromosome 5 (group S3). Values are means ± SEM (*n* = 4–6 biological replicates). There was a significant effect of time of day on the expression of *TaRbcS* (ANOVA, *P* < 0.05). The times of day which are significantly different are marked with asterisks [Tukey’s honestly significant difference (HSD) mean-separation test, *P < 0.05 and **P < 0.01]

The relative gene expression of the Rubisco subunits, *TarbcL* and *TaRbcS*, also fluctuated during the diel cycle; however, these fluctuations were only significant for the small subunit, *TaRbcS*, which showed lowest expression at 4 h into the photoperiod and highest expression at 10 h into the photoperiod (Fig. 2). The relative expression levels of both Rubisco subunits, *TarbcL* and *TaRbcS*, did not show a consistent trend during the dark phase, remaining mostly stable.

### TaRca-β was more abundant than TaRca-α and the isoform ratio remained unchanged during the diel cycle

**Fig. 3:**
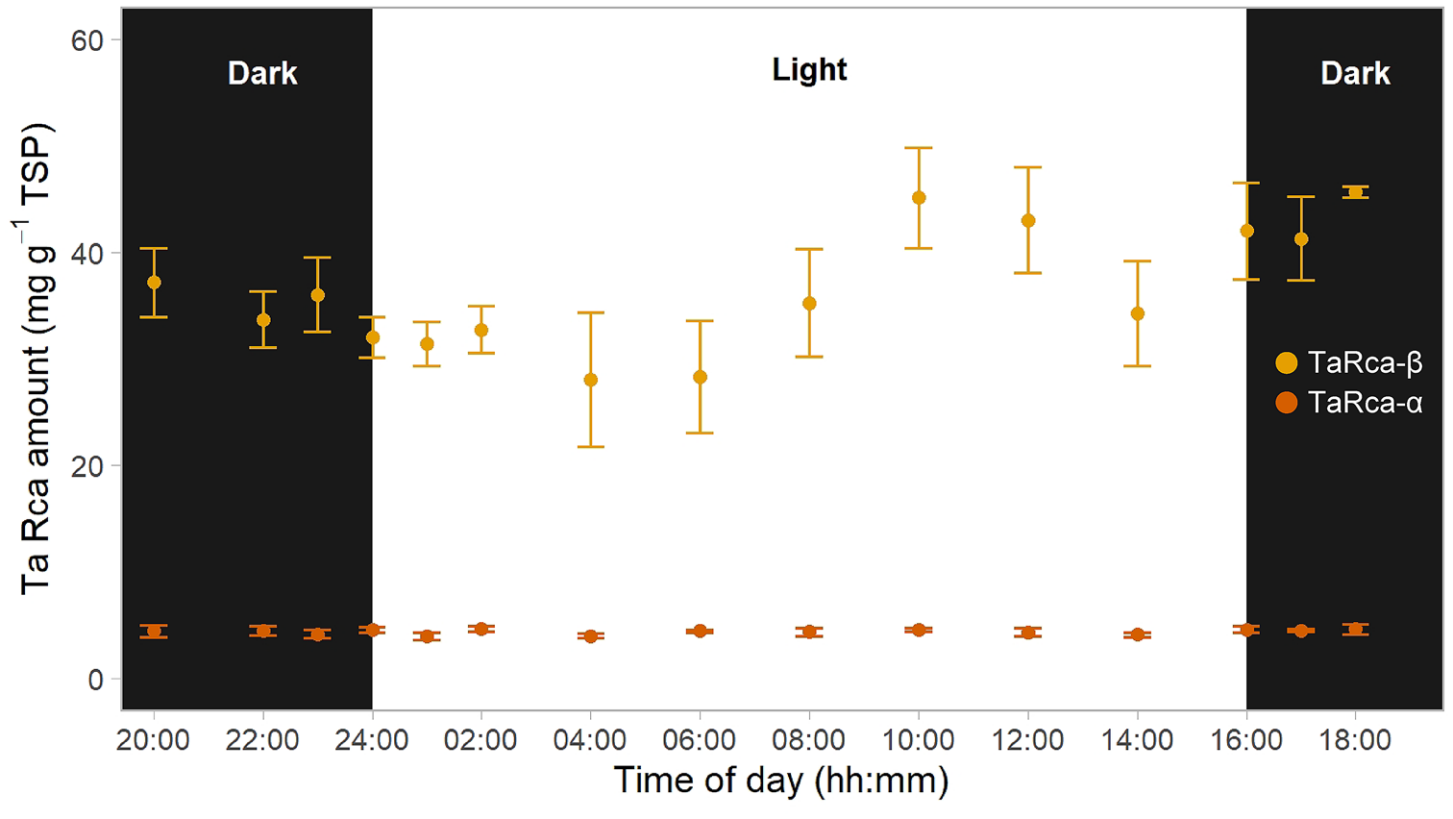
TaRca-β and TaRca-α protein amounts throughout the diel cycle in young fully expanded leaves of wheat. Samples were taken from individual plants 38 days after planting (vegetative stage) at the indicated times during the night (“Dark”, black background) and day (“Light”, white background). Rca amount was estimated by reference to a calibration curve prepared with increasing amounts of purified TaRca. Values are means ± SEM (*n* = 4–6 biological replicates). There were no significant differences between time of day and TaRca amount (ANOVA, *P* > 0.05)

The protein abundance of TaRca-β and TaRca-α in young fully expanded leaves of wheat plants throughout the diel cycle was estimated by immunoblotting. Because of the barely detectable transcript abundance of TaRca1-β (Fig. 1), we assumed that the antibody reactivity to the shorter Rca-β represents mainly TaRca2-β. TaRca-β was much more abundant than TaRca-α during the entire diel cycle (Fig. 3). Although the TaRca-β amounts appeared highest at 10 h into the photoperiod, there were no significant differences between the time points at which measurements were taken during the diel cycle. Similarly, TaRca-α remained constant during the entire diel cycle (Fig. 3). The protein ratio between the isoforms TaRca-α and TaRca-β was 12.5 ± 0.5% throughout the diel cycle (Fig. S3).

### Rubisco abundance decreased during the dark period

**Fig. 4:**
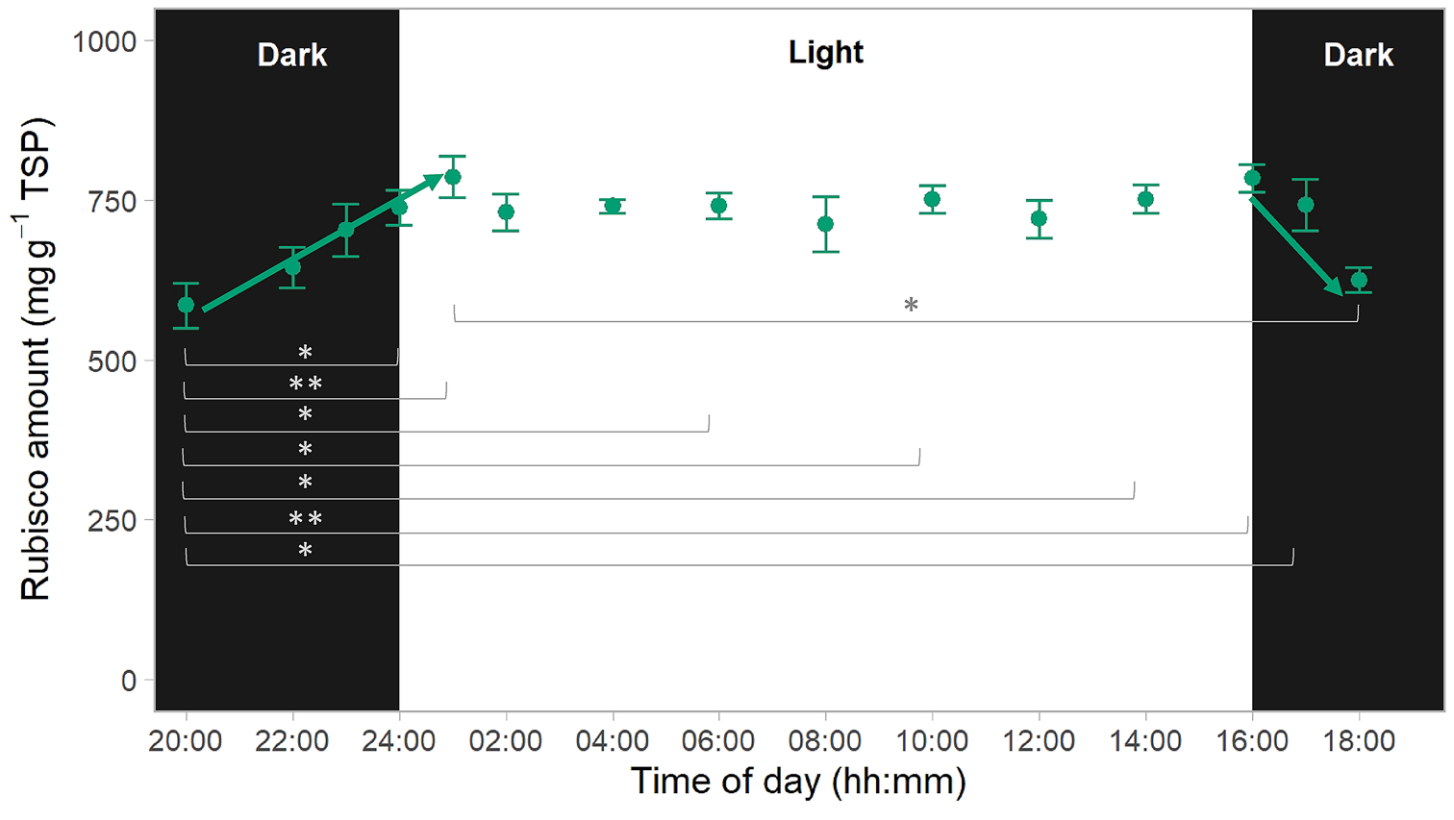
Rubisco amount throughout the diel cycle in young fully expanded leaves of wheat. Samples were taken from individual plants 38 days after planting (vegetative stage) at the indicated times during the night (“Dark”, black background) and day (“Light”, white background). Rubisco amount was estimated by reference to a calibration curve prepared with increasing amounts of purified enzyme and based on density of the large subunit (TarbcL). Values are means ± SEM (*n* = 4–6 biological replicates). The times of day which are significantly different are marked with asterisks [Tukey’s honestly significant difference (HSD) mean-separation test, *P < 0.05 and **P < 0.01]. Arrows mean significant, positive and negative, correlations between Rubisco amount and time of the day (Pearson correlation analysis, *P* < 0.05)

The amount of Rubisco protein estimated by SDS-PAGE based on the abundance of the large subunit relative to total soluble protein remained constant throughout the photoperiod but decreased in the dark period (Fig. 4). Rubisco abundance was lowest 4 h into the dark period, with a significant difference between the value at the middle of the dark period (20:00 h) and most of the values during the photoperiod. Moreover, there was a significant decrease in Rubisco abundance in the first half of the dark period followed by a significant increase in the second half of the photoperiod (Fig. 4). The higher level of abundance observed at the start of the photoperiod was maintained during the light phase.

### TaRca-β exhibited a time-lag between gene expression and protein amount

To estimate the maximum gene expression and protein amount for Rca isoforms and Rubisco, and the corresponding times at which the maximum values are obtained, a generalised additive model (GAM) was fitted to the experimental data (Figs. S4, S5). Based on the model predictions, *TaRca2-β* and *TaRca2-α* had their maximum relative gene expression values (117.1 and 85.1 NRQ, respectively) at 09:15 h into the photoperiod (Table 1). By comparison, the maximum predicted amount of TaRca-β protein was 42.5 mg g^−1^ TSP at 10:45 h (1.5 h after the peak in gene expression), and there was no peak for TaRca-α protein during the photoperiod.

**Table 1:** Predicted values for Rca and Rubisco transcript and protein abundance in wheat

Isoform/subunit	Predicted value	Time of day (hh:mm)	*P* value
*TaRca1-β* (mean NRQ)	0.002 (Mean)	NA	0.583
*TaRca2-β* (mean NRQ)	117.1 (Max)	09:15	< 0.001***
*TaRca2-α* (mean NRQ)	85.1 (Max)	09:15	< 0.001***
TaRca-β (mg g^−1^ TSP)	42.5 (Max)	10:45	< 0.01**
TaRca-α (mg g^−1^ TSP)	4.35 (Mean)	NA	0.411
*TarbcL* (mean NRQ)	446.1 (Mean)	NA	0.594
*TaRbcS* (mean NRQ)	185.6 (Max)	10:30	< 0.05*
TarbcL (mg g^−1^ TSP)	770.0 (Max)	01:00	< 0.001***

The maximum and mean abundances, the times at which these occurred and the respective *P* values (*P<0.05, **P<0.01 and ***P<0.001) were estimated from the best-fit models applied to describe the diurnal response of Rca and Rubisco gene expression and protein amounts (Figs. S4, S5; Table S2)

Conversely, for Rubisco, there was a predicted peak in the large subunit protein amount at 01:00 h into the photoperiod, with no corresponding peak in gene expression, which remained constant throughout the light period (Table 1). These results show a lag between gene expression and protein abundance for TaRca-β, and a generalised lack of correlations between gene expression and protein abundance of Rubisco and Rca during the diel cycle (Fig. S6).

## Discussion

Rubisco activase (Rca), a molecular chaperone required to maintain Rubisco activity, occurs in diverse isoforms. In wheat, three isoforms have been described that are known to differ in their redox-sensitivity (by inference from results with *Arabidopsis*; Zhang and Portis, 1999; Zhang et al. 2001) and inhibition by ADP (Perdomo et al. 2019; Degen et al. 2020). Here, we show that the relative abundance of the redox-sensitive TaRca-α and redox-insensitive TaRca-β isoforms remains constant in the leaves of wheat plants grown under controlled conditions throughout the entire diel cycle. TaRca-α isoform is less abundant, representing 85% of the redox-insensitive TaRca-β at the transcript level and 12.5% at the protein level.

*Rca* gene expression has been reported to respond to organ-specific signals, light availability and the circadian clock in a species-specific manner (Orozco and Ogren 1993; Watillon et al. 1993; Liu et al. 1996; Chao et al. 2014). The expression of the wheat Rca isoforms *TaRca2-α* and *TaRca2-β*, encoded by the highly expressed *TaRca2* gene, increased during the first half of the photoperiod, with a peak at 8–10 h after the start of the 16 h photoperiod. The expression of *TaRca1-β* isoform was extremely low, in agreement with previous reports (Scafaro et al. 2019a; Degen et al. 2021), and remained low throughout the entire diel cycle (Fig. 1). *Rca* expression peaked earlier in the day for other species previously characterised: 3 h after the start of a 14 h photoperiod in rice (To et al. 1999), 6 h after the start of a 14 h photoperiod in *Arabidopsis* (Pilgrim and McClung 1993), 2 h after the start of a 14 h photoperiod in soybean (Chao et al. 2014), and 2 h after the start of a 16 h photoperiod in apple (Watillon et al. 1993). It is also possible that the relative expression of *Rca* isoforms in different species may vary with growth conditions, plant age and developmental stage.

The expression of Rubisco large (*rbcL*) and small (*RbcS*) subunits is controlled by different internal and external factors such as light signals and temperature (Rodermel et al. 1996; Spreitzer 2003; Recuenco-Muñoz et al. 2015). The expression of *TarbcL* remained constant during the diel cycle, while *TaRbcS* transcripts abundance was highest 10 h into the photoperiod (Fig. 2). Of note is that the primers used were specific to the *TaRbcS* genes in chromosome 5 (group S3). As described by Degen et al. (2021), *TaRbcS* genes can be grouped according to their sequence similarity, with gene groups S1 and S2 in chromosome 2 and group S3 in chromosome 5. In the same study, the abundance of *TaRbcS2* and *TaRbcS3* transcripts was comparable and higher than *TaRbcS1* in wheat leaves that were sampled only at 4 h after the start of a 16 h photoperiod. The results presented here are in agreement with previous studies reporting that the Rubisco *RbcS* is regulated by the circadian rhythm, while the *rbcL* is not (Pilgrim and McClung 1991; Cheng et al. 1998; Recuenco-Muñoz et al. 2015). In *Arabidopsis* the peak in expression of *RbcS* was reported earlier in the light phase, i.e. 1 h after the start of a 14 h photoperiod, and in *C. reinhardtii* and tomato *RbcS* expression remained largely constant throughout the diel cycle (Martino-Catt and Ort 1992; Pilgrim and McClung 1993; Cheng et al. 1998; Recuenco-Muñoz et al. 2015), suggesting a species-specific pattern. Whether *TaRbcS1* and *TaRbcS2* show a peak of expression at the middle of the photoperiod as observed for *TaRbcS3* remains to be determined.

Rubisco protein abundance was highest at the start of the photoperiod and remained constant during the light phase. The CO_2_-fixing enzyme represents a large fraction of the total soluble protein (TSP) in wheat leaves, both in plants grown under controlled conditions (Fig. 4) and in the field (Carmo-Silva et al. 2015, 2017). The decrease in Rubisco abundance observed during the night period in the present study suggests significant degradation of the enzyme in the first 4 h, followed by synthesis prior to the start of the photoperiod. The extent of Rubisco degradation cannot be accurately determined by SDS-PAGE and requires further investigation using more robust methods, including CABP-binding (Ferreira et al. 2000; Whitney and Sharwood 2014). Previous work has suggested that Rubisco is degraded at a slow rate both in rice (Mae et al. 1983; Suzuki et al. 2001; Irving and Robinson 2006) and in *Arabidopsis* (Li et al. 2017; Tivendale et al. 2020). Esquível et al. (1998) found the rate of Rubisco degradation to be species-specific; and it is likely that the degradation rate changes with leaf and plant age, as well as with environmental conditions. Importantly, large daily fluctuations of the most abundant leaf protein represent a significant cellular energetic burden, and Rubisco abundance has been shown to impact wheat grain yields (e.g. Lobo et al. 2019). Thus, further investigation is warranted into the rates of Rubisco synthesis and degradation, and the regulation of Rubisco protein abundance and turnover.

Variation in Rubisco activity during the photoperiod is more likely to be controlled via regulation of its activity than protein abundance (Li et al. 2020; Davies and Griffiths 2012). The molecular chaperone Rca is a key player in the regulation of Rubisco activity. Modelling of Rca isoform protein amounts suggested a peak in the abundance of TaRca-β at 10:45 h into the photoperiod (Table 1), while TaRca-α abundance remained unchanged throughout the diel cycle. In tomato, Rca abundance peaked 2 h before the start of the photoperiod and was lowest at the middle of the photoperiod (Martino-Catt and Ort 1992). The ratio between total Rca and Rubisco abundance remained largely constant during the photoperiod, suggesting sufficient amount of the molecular chaperone to maintain Rubisco activity through the release of inhibitory compounds from active sites.

The relative abundance of the different Rca isoforms differs among species. In *Arabidopsis*, *Camelina* and spinach, equal amounts of Rca-α and Rca-β are present, while rice and soybean accumulate much more Rca-β than Rca-α (Salvucci et al. 1987; Fukayama et al. 2012; Chao et al. 2014; Scafaro et al. 2016). Similarly, in wheat, TaRca-β is much more abundant than TaRca-α (Law and Crafts-Brandner 2001; Degen et al. 2021), and the Rca-α/β ratio remained constant during the whole diel cycle (12.5%, Fig. S3). The three wheat Rca isoforms have been shown to differ in the sensitivity of Rubisco activation activity to inhibition by ADP (Perdomo et al. 2019; Scafaro et al. 2019b), as well as in response to temperature (Scafaro et al. 2019a; Degen et al. 2020). It would be conceivable that plants might up-regulate the abundance of one or another isoform in adaptation to the prevailing environment. Accordingly, the Rca-α/β ratio has been reported to increase in *Brachypodium distachyon* plants under drought and salinity stress (Bayramov and Guliyev 2014), and the relative abundance of TaRca1-β increased in wheat at high temperature (Law and Crafts-Brandner 2001; Degen et al. 2021).

Coordination between gene expression and protein translation enables physiological responses to various environmental stimuli, essential for successful plant growth and reproduction (Grabsztunowicz et al. 2017). However, for both Rca isoforms and Rubisco, there was a mismatch between the time of day corresponding to the predicted maximum levels of transcript abundance and protein abundance (Table 1). While for TaRca-β the maximum protein abundance was predicted to occur 1.5 h after the predicted peak of transcript abundance, for TaRca-α and for Rubisco there was a peak for transcript abundance and not for protein abundance or vice-versa. Moreover, the ratio TaRca-α/TaRca-β was 85% at the transcript level and 12.5% at the protein level, and there was no significant correlation between gene expression and protein abundance for Rca and Rubisco during the diel cycle (Fig. S6). These findings agree with previous studies in wheat and other species under control and stress conditions, suggesting that Rca is regulated at either the translational level by mRNA silencing or at post-translational level by more rapid turnover of the protein (Law and Crafts-Brandner 2001; DeRidder et al. 2012; Bayramov and Guliyev 2014). A lack of correlation between gene expression and protein synthesis has also been reported for Rubisco in *Arabidopsis*, tobacco, rice and *C. reinhardtii* (Pilgrim and McClung 1993; Rodermel et al. 1996; Wang and Wang 2011; Recuenco-Muñoz 2015), again suggesting that abundance of the CO_2_-fixing enzyme might be regulated post-transcriptionally (Rodermel et al. 1996; Law and Crafts-Brandner 2001; Houtz and Portis 2003).

In summary, the results presented here show that the redox-sensitive TaRca-α isoform is less abundant than the redox-insensitive TaRca-β isoform, the difference in abundance is more pronounce at the protein level (12.5% α/β) than at the transcript level (85% α/β), and the ratio TaRca-α/TaRca-β remains unchanged throughout the diel cycle. These results, combined with the lack of correlation between transcript and protein abundance for both Rca and Rubisco, suggest that the abundance of both enzymes and their isoforms is post-transcriptionally regulated.

## Supplementary Information

Below is the link to the electronic supplementary material.

Supplementary file1 (XLSX 26 kb)

Supplementary file2 (DOCX 533 kb)
