# Supplementary material for: The relative abundance of wheat Rubisco activase isoforms is post-transcriptionally regulated
Source: Photosynth Res. 2021 Apr 1;148(1):47–56. doi: 10.1007/s11120-021-00830-6 (PMC8154801; doi:10.1007/s11120-021-00830-6)
Supplement: Supplementary file 2 — Supplementary file2 (DOCX 533 kb) [file 11120_2021_830_MOESM2_ESM.docx]

**The relative abundance of wheat Rubisco activase isoforms is post-transcriptionally regulated**

Juan Alejandro Perdomo^1*^, Peter Buchner^2^, Elizabete Carmo-Silva^1^

^1^Lancaster Environment Centre, Lancaster University, Lancaster, LA1 4YQ, UK.

^2^Plant Biology and Crop Science Department, Rothamsted Research, Harpenden AL5 2JQ, U.K.

*Contact E-mail: [alejandro.perdomo@lancaster.ac.uk](mailto:alejandro.perdomo@lancaster.ac.uk)

**Fig. S1**. Light regime and sampling times.

**Fig. S2.** TaRca and Rubisco quantification.

**Fig. S3.** Rca-α and -β ratios.

**Fig. S4**. Model predictions for Rca gene expression and protein amount.

**Fig. S5**. Model predictions for Rubisco gene expression and protein amount.

**Fig. S6.** Relative gene expression and protein correlations.

**Table S1.** Sequences of qRT-PCR primer pairs used for measuring Rca and Rubisco gene expression in wheat plants.

**Table S2.** Modelling the diurnal response of Rca and Rubisco gene expression and protein abundance in wheat.

**Dark Light Dark**

**Figure S1**. **Light regime and sampling times**. The photosynthetic photon flux density (PPFD) throughout the day (blue line) and the times at which samples were taken (red symbols).


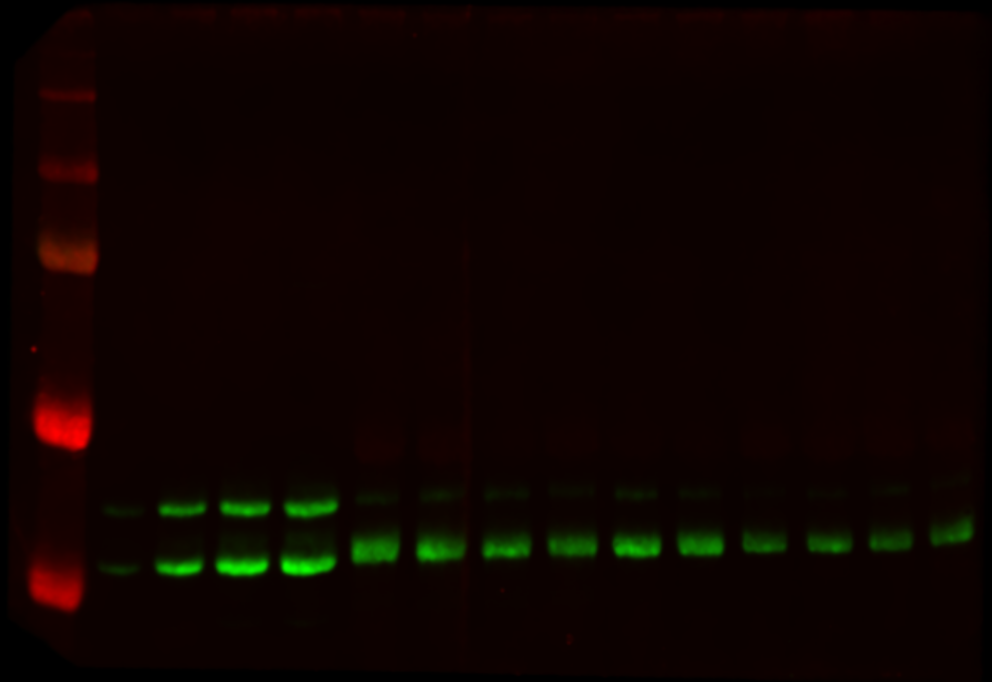


Calibration curve Samples

0.01 0.05 0.1 0.15


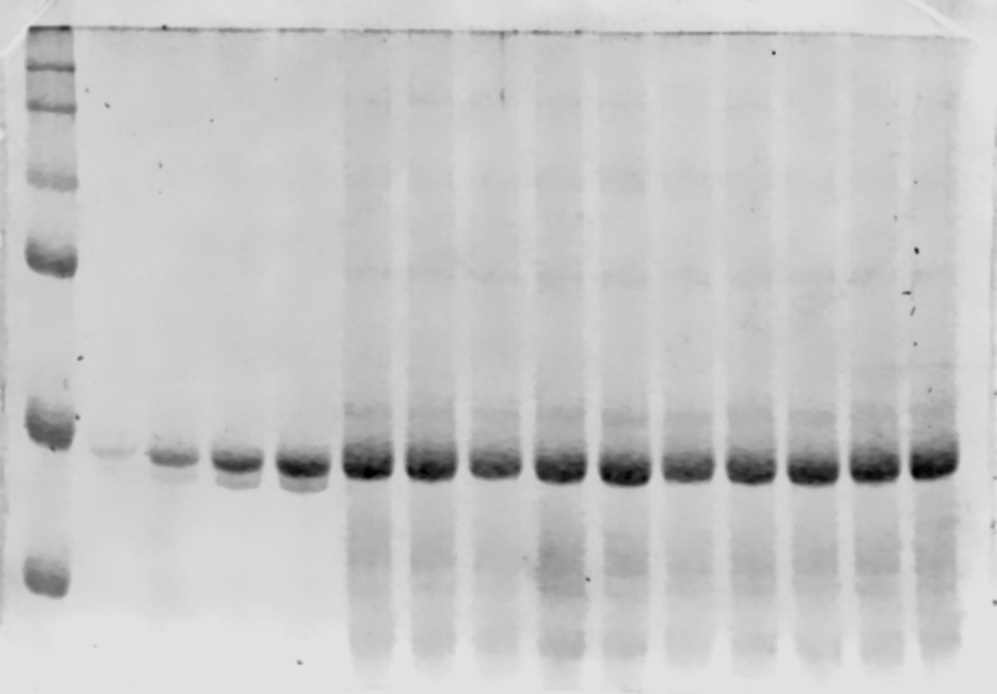


TaRca-α

TaRca-β

Calibration curve Samples

0.1 0.5 1.0 1.5

Rubisco LSU

**Figure S2. TaRca and Rubisco quantification**. Wheat Rca isoforms (TaRca-α and TaRca-β) and Rubisco large subunit (LSU) quantification by immunoblotting and Coomassie staining, respectively. Rca amount was estimated by reference to a calibration curve prepared with increasing amounts (0.01, 0.05, 0.1 and 0.15 µg) of purified TaRca. Rubisco amount was estimated by reference to a calibration curve prepared with increasing amounts (0.1, 0.5, 1.0 and 1.5 µg) of purified enzyme.


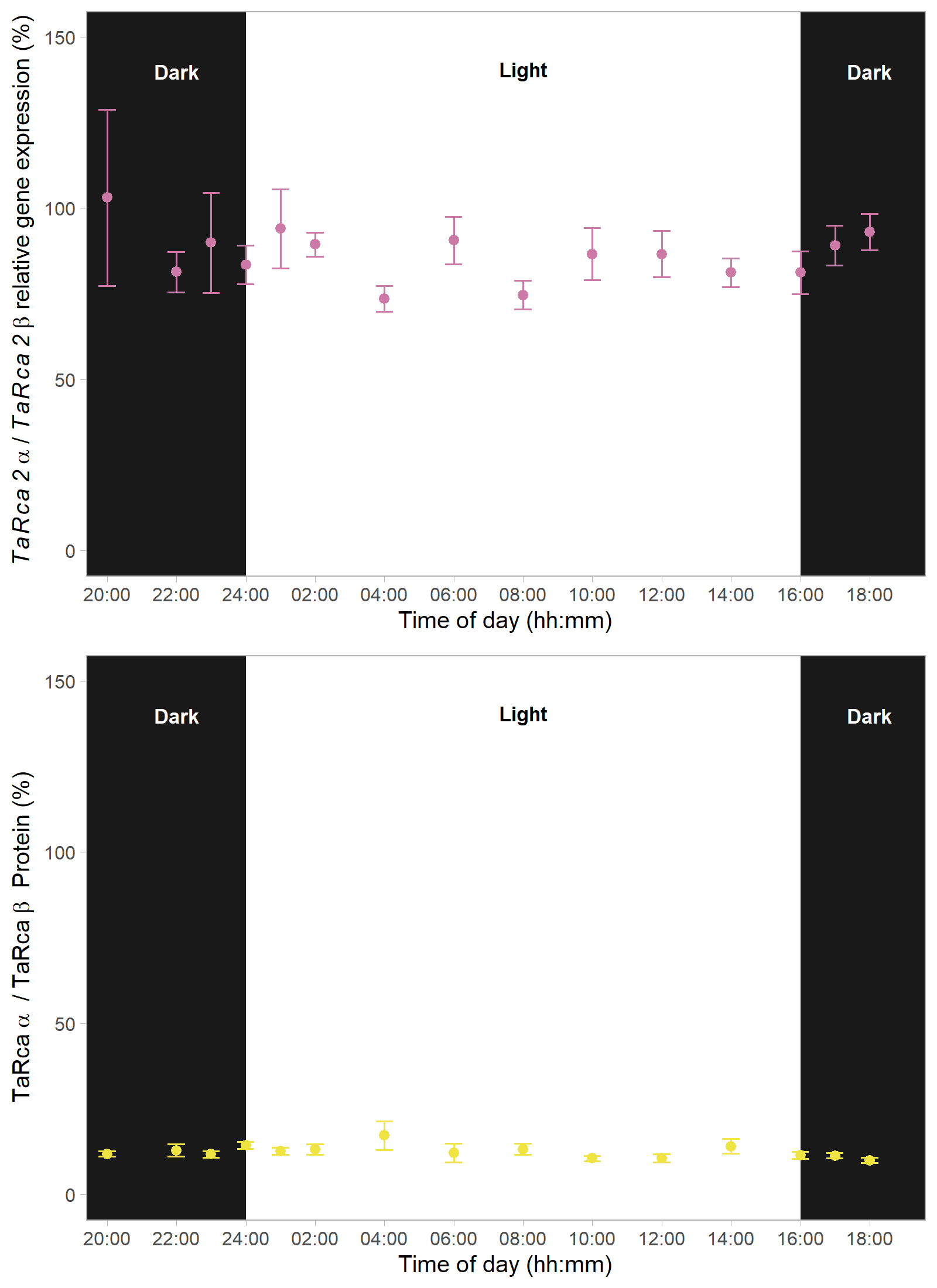


**Figure S3. Rca-α to Rca-β ratio**. Ratio between *TaRca2-α* and *TaRca2-β* relative gene expression and TaRca-α and TaRca-β amounts throughout the diel cycle. Values are means ± SE (n = 4-6 biological replicates). There were no significant differences in Rca isoform ratios between different times of the day (ANOVA, *P* > 0.05), with overall mean values of 85±3% (gene expression) and 12.5±0.5% (protein).


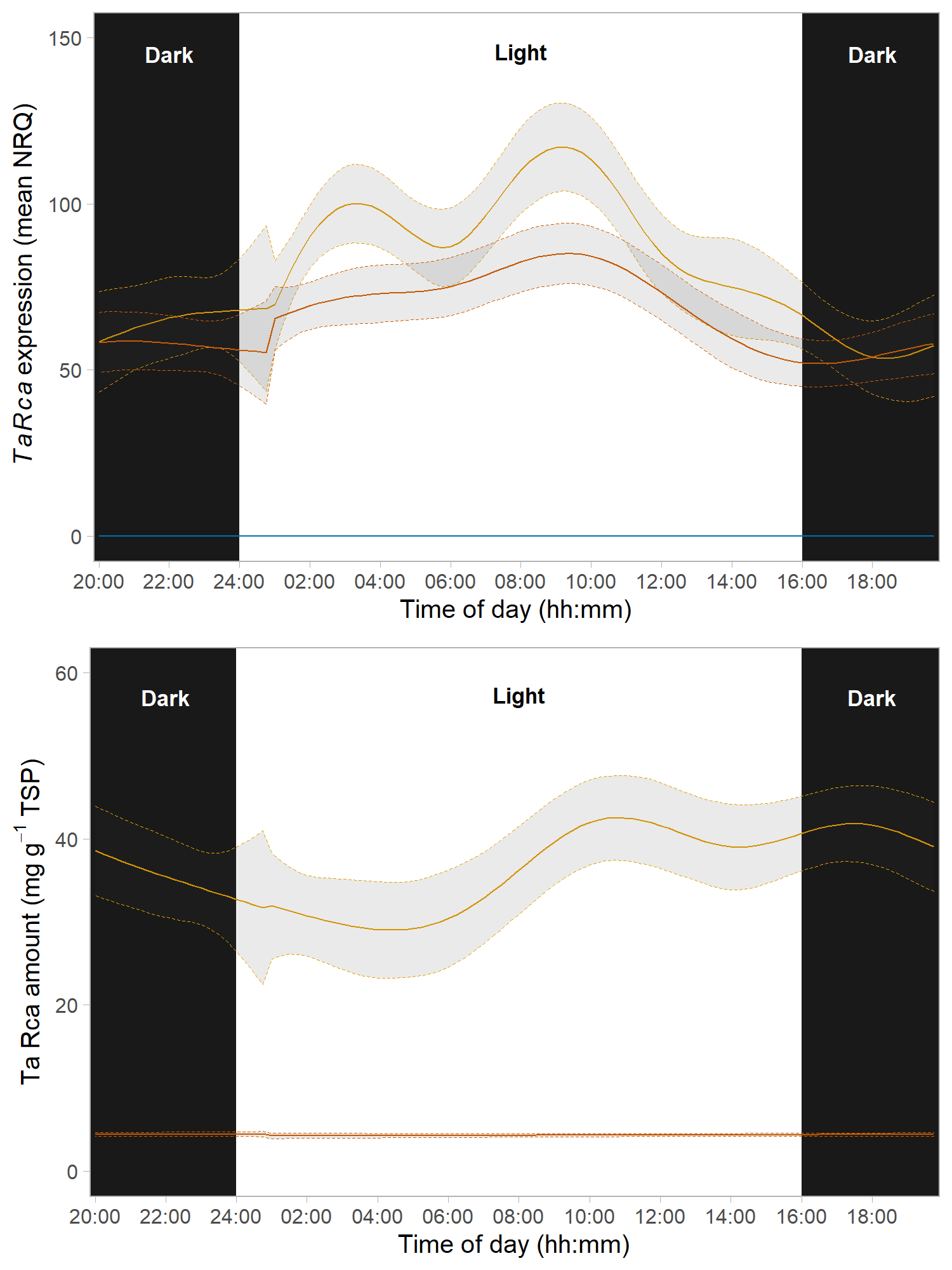


TaRca-β

TaRca-α

*TaRca1-β*

*TaRca2-β*

*TaRca2-α*

**Figure S4.** **Model predictions for Rca relative gene expression and protein amount**. *TaRca1-β*, *TaRca2-β*, *TaRca2-α* expression and TaRca-β, TaRca-α amounts throughout the diel cycle. Lines represent the best-fit for each Rca isoform (AIC, Table S2) and coloured areas within dashed lines denote the 95% confidence interval for each fit.


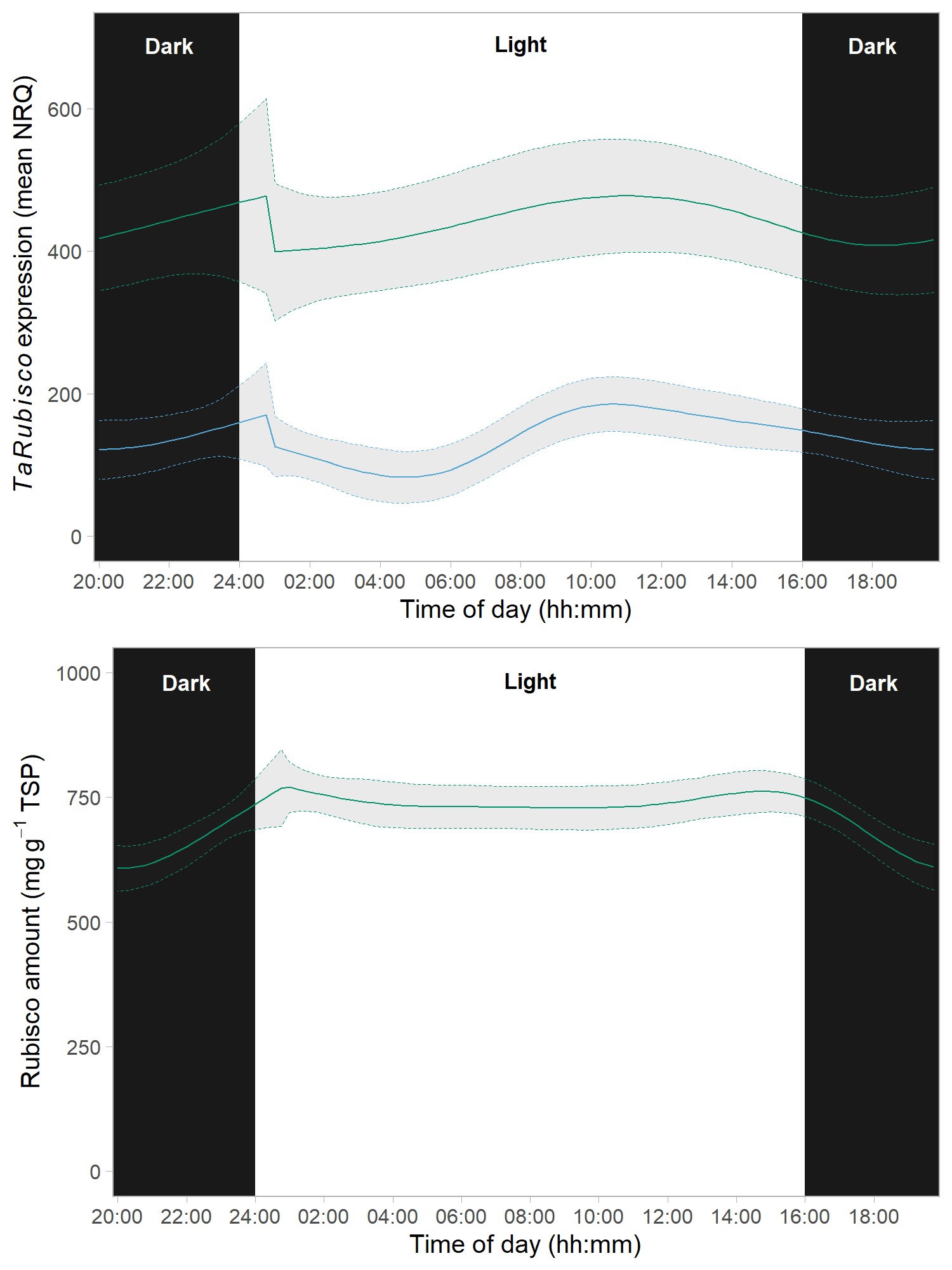


Rubisco LSU

*TarbcL*

*TaRbcS*

**Figure S5.** **Model predictions for Rubisco relative gene expression and protein amount.** *TarbcL,* *TaRbcS* expression and Rubisco large subunit (LSU) amount throughout the diel cycle. Lines represent the best-fit for each Rubisco subunit (AIC, Table S2) and coloured areas within dashed lines denote the 95% confidence interval for each fit.

**Figure S6.** **Relative gene expression and protein correlations.** Relative gene expression and protein amount correlations for *TaRca2-β, TaRca2-α* and Rubisco large subunit (LSU)*.* Samples were taken from individual plants (vegetative stage) during the night (black symbols) and day (white symbols). There was no significant correlation between gene expression and protein amount in either of the two Rca isoforms and Rubisco (Pearson, *P* > 0.05).

**Table S1.** **Sequences of qRT-PCR primer pairs used for measuring Rca and Rubisco gene expression in wheat plants**.

| **Gene** | **Gene ID** | **Primer** | **Primer sequences** |
| --- | --- | --- | --- |
| *Rca1β* | TraesCS4A02G177600 TraesCS4B02G140200  TraesCS4D02G134900 | Forward  Reverse | GGG TCG GCG AGA TCG GCG T  CCA GCA TGT GGC CGT ACT CCA TG |
| *Rca2β* | TraesCS4A02G177500  TraesCS4B02G140300  TraesCS4D02G135000 | Forward  Reverse | CCA TAC ACA CCC ACC ATC TCT TGC  TGT AAA GGC AGC TCC CGT CGT |
| *Rca2α* |  | Forward  Reverse | CCT TCT ACG GTA AAG GGG CAC AG  TGT AAA GGC AGC TCC CGT CGT |
| *rbcL* | AY328025 chloroplast gene | Forward  Reverse | ACC ATT TAT GCG CTG GAG AGA CC  CAA GTA ATG CCC CTT GAT TTC ACC |
| *RbcS* | 100% specific for all chr 5A/B/D | Forward  Reverse | GGA TTC GAC AAC ATG CGY CAG G  ATA TGG CCT GTC GTG AGT GAG C |
| *Succinate DHG* | TraesCS2A02G220800  TraesCS2B02G246400  TraesCS2D02G226500 | Forward  Reverse | TTT GCT CTC CGT GGT GCC TTT GG  GAA GAT GTG TAG CTC CTT GCT TGC |
| *Actin* | TraesCS5A02G124300  TraesCS5B02G124100  TraesCS5D02G132200 | Forward  Reverse | ATC TCG AAG GGY GAG TAT GAY GAG  AGA AGA CCC AGA CAA CTC GCA AC |

**Table S2. Modelling the diurnal response of Rca and Rubisco gene expression and protein abundance in wheat.** The model providing the best fit to the data is highlighted in bold and was selected according to the lowest AIC score (Akaike information criterion) calculated according to (Akaike, 1974) using the AIC function in R. Models were applied to the full dataset shown in Figs. 1, 2, 3 and 4, using individual data points.

| Protein | **Gene Expression** | | | **Abundance** | | |
| --- | --- | --- | --- | --- | --- | --- |
|  | Model | degrees of freedom (K) | AIC score | Model | degrees of freedom (K) | AIC score |
| Rca 1β | 2^nd^ order polynom | 4.0 | -742.2 | - | - | - |
|  | 3^rd^ order polynom | 5.0 | -743.2 | - | - | - |
|  | 4^th^ order polynom | 6.0 | -743.1 | - | - | - |
|  | 5^th^ order polynom | 7.0 | -741.4 | - | - | - |
|  | **GAM** | **4.6** | **-743.8** | - | - | - |
| Rca 2β | 2^nd^ order polynom | 4.0 | 642.3 | 2^nd^ order polynom | 4.0 | 617.1 |
|  | 3^rd^ order polynom | 5.0 | 622.5 | 3^rd^ order polynom | 5.0 | 617.2 |
|  | 4^th^ order polynom | 6.0 | 624.4 | 4^th^ order polynom | 6.0 | 617.7 |
|  | 5^th^ order polynom | 7.0 | 625.3 | 5^th^ order polynom | 7.0 | 619.0 |
|  | **GAM** | **10.2** | **613.9** | **GAM** | **7.8** | **614.9** |
| Rca 2α | 2^nd^ order polynom | 4.0 | 617.4 | 2^nd^ order polynom | 4.0 | 207.7 |
|  | 3^rd^ order polynom | 5.0 | 607.4 | 3^rd^ order polynom | 5.0 | 209.7 |
|  | 4^th^ order polynom | 6.0 | 609.4 | 4^th^ order polynom | 6.0 | 211.6 |
|  | 5^th^ order polynom | 7.0 | 607.1 | 5^th^ order polynom | 7.0 | 213.3 |
|  | **GAM** | **7.9** | **601.1** | **GAM** | **3.0** | **206.1** |
| rbcL | 2^nd^ order polynom | 4.0 | 902.9 | 2^nd^ order polynom | 4.0 | 995.3 |
|  | 3^rd^ order polynom | 5.0 | 903.8 | 3^rd^ order polynom | 5.0 | 996.7 |
|  | 4^th^ order polynom | 6.0 | 903.7 | 4^th^ order polynom | 6.0 | 986.4 |
|  | 5^th^ order polynom | 7.0 | 903.9 | 5^th^ order polynom | 7.0 | 984.8 |
|  | **GAM** | **5.3** | **901.5** | **GAM** | **8.4** | **980.2** |
| RbcS | 2^nd^ order polynom | 4.0 | 839.9 | - | - | - |
|  | 3^rd^ order polynom | 5.0 | 841.9 | - | - | - |
|  | 4^th^ order polynom | 6.0 | 833.9 | - | - | - |
|  | 5^th^ order polynom | 7.0 | 835.4 | - | - | - |
|  | **GAM** | **7.5** | **832.1** | - | - | - |
